# Supplementary material for: Depicting Fecal Microbiota Characteristic in Yak, Cattle, Yak-Cattle Hybrid and Tibetan Sheep in Different Eco-Regions of Qinghai-Tibetan Plateau
Source: Microbiol Spectr. 2022 Jul 12;10(4):e00021-22. doi: 10.1128/spectrum.00021-22 (PMC9430443; doi:10.1128/spectrum.00021-22)

Supplementary

TABLE S1 Population information of samples

Groups	Gender		Age (year old)		
	Male	Female	≤2	3~4	≥5
Yak-TD	28	22	48	2	0
Yak-CK	17	16	13	14	6
Yak-DQ	4	23	17	10	0
Yak-MK	12	28	21	16	3
Cattle-CK	5	17	15	3	4
Cattle-DQ	0	4	4	0	0
YC-DQ	30	0	0	30	0
TS-TD	12	38	29	20	1
TS-MK	12	25	17	20	0
TS-CK	6	35	35	3	3

FIG S1 The different alpha diversity between GIN infected and non-infected group. Alpha diversity based on Shannon and Chao1 indexes of fecal bacteria. *t*-test was used for statistical analysis between Bos-N and Bos-P, Sheep-N and Sheep-P, * means P<0.05. Bos-N and Bos-P, feces of Bos without/with GIN infection; Sheep-N and Sheep-P, feces of Sheep without/with GIN infection.

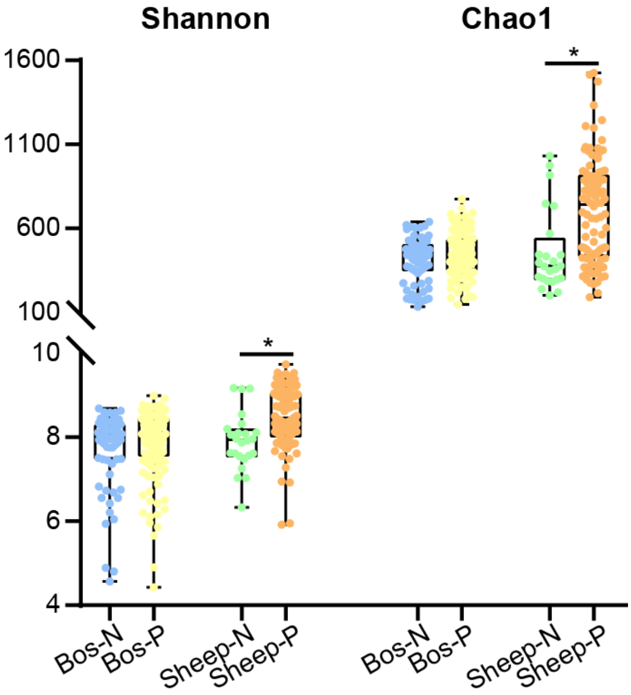

FIG S2 Beta diversity of the fecal bacteria communities among cattle, yaks and Tibetan sheep in different age and gender. Weighted Unifrac distance-based principal coordinate analysis (PCoA) plot for (a) Yak-CK, feces of yaks in Caka; (b) Cattle-CK, feces of cattle in Caka; (c) TS-CK, feces of Tibetan Sheep in Caka; (d) TS-TD, feces of Tibetan Sheep in Tongde, respectively. F, female; M, male.

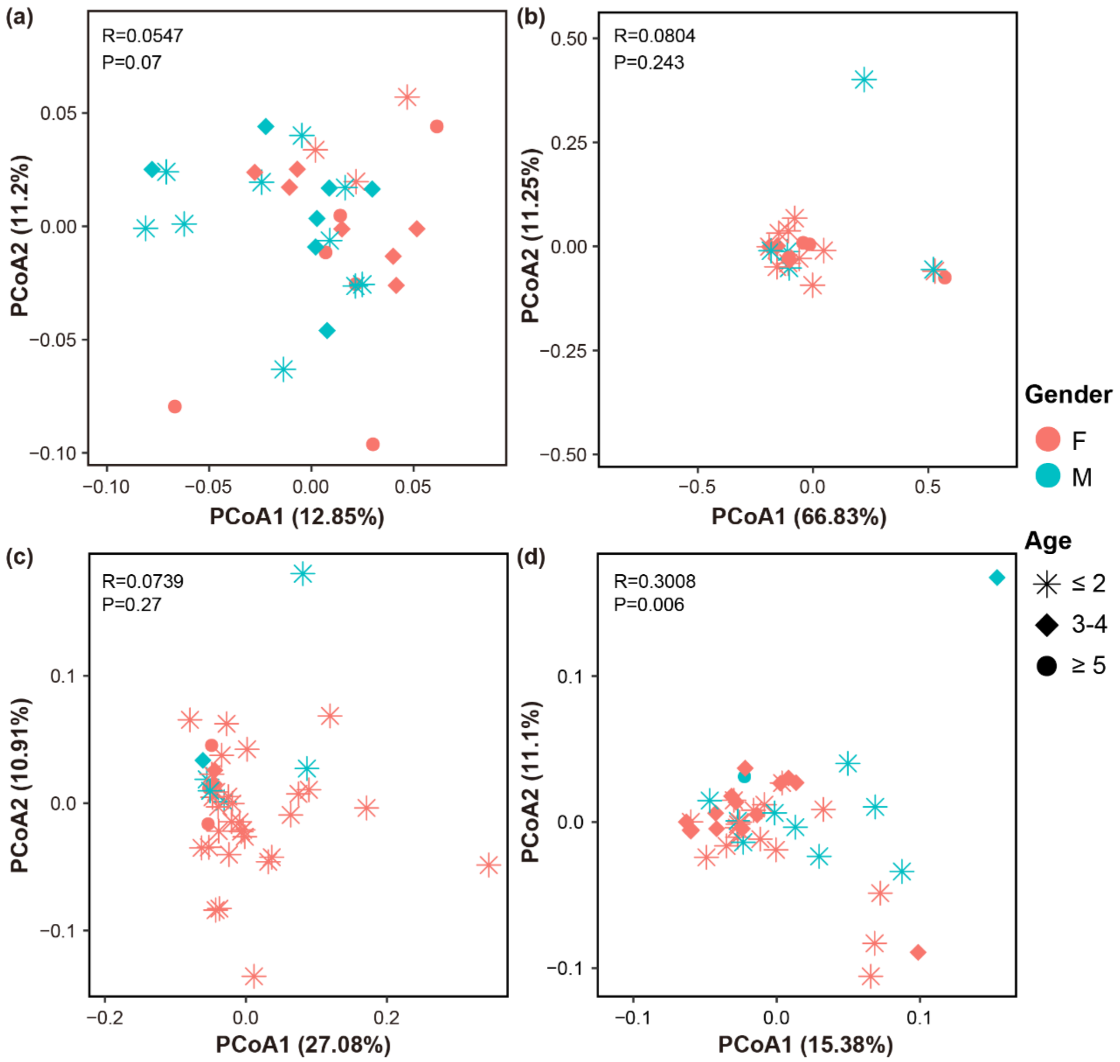

FIG S3 The relative abundance of bacterial phyla among cattle, yaks and yak-cattle hybrids.

The top 4 phyla in relative abundance are *Firmicutes*, *Bacteroidetes*, *Proteobacteria*, *Verrucomicrobia*. The significance was signed by lowercase letters (ANOVA).

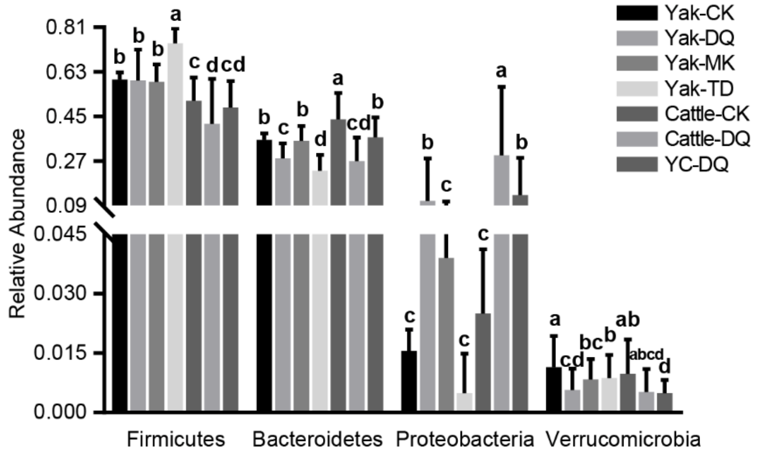

FIG S4 The relative abundance of bacterial phyla among Tibetan sheep from different regions.

The top 4 phyla in relative abundance are *Firmicutes*, *Bacteroidetes*, *Proteobacteria*, *Verrucomicrobia*. The significant difference was signed by lowercase letters (ANOVA).

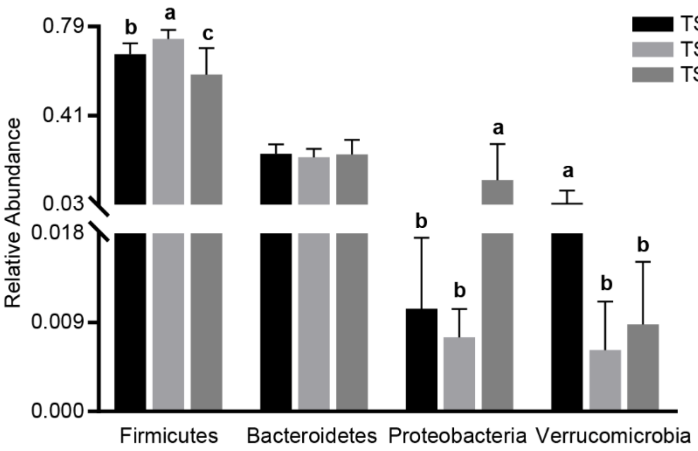

FIG S5 The bacterial taxonomic composition of GIN negative and positive groups. The bacterial taxonomic composition at (a) phylum level of GIN negative and positive groups, (b) genus level of GIN negative and positive groups. Bos-N and Bos-P, feces of Bos without/with GIN infection; Sheep-N and Sheep-P, feces of Sheep without/with GIN infection.

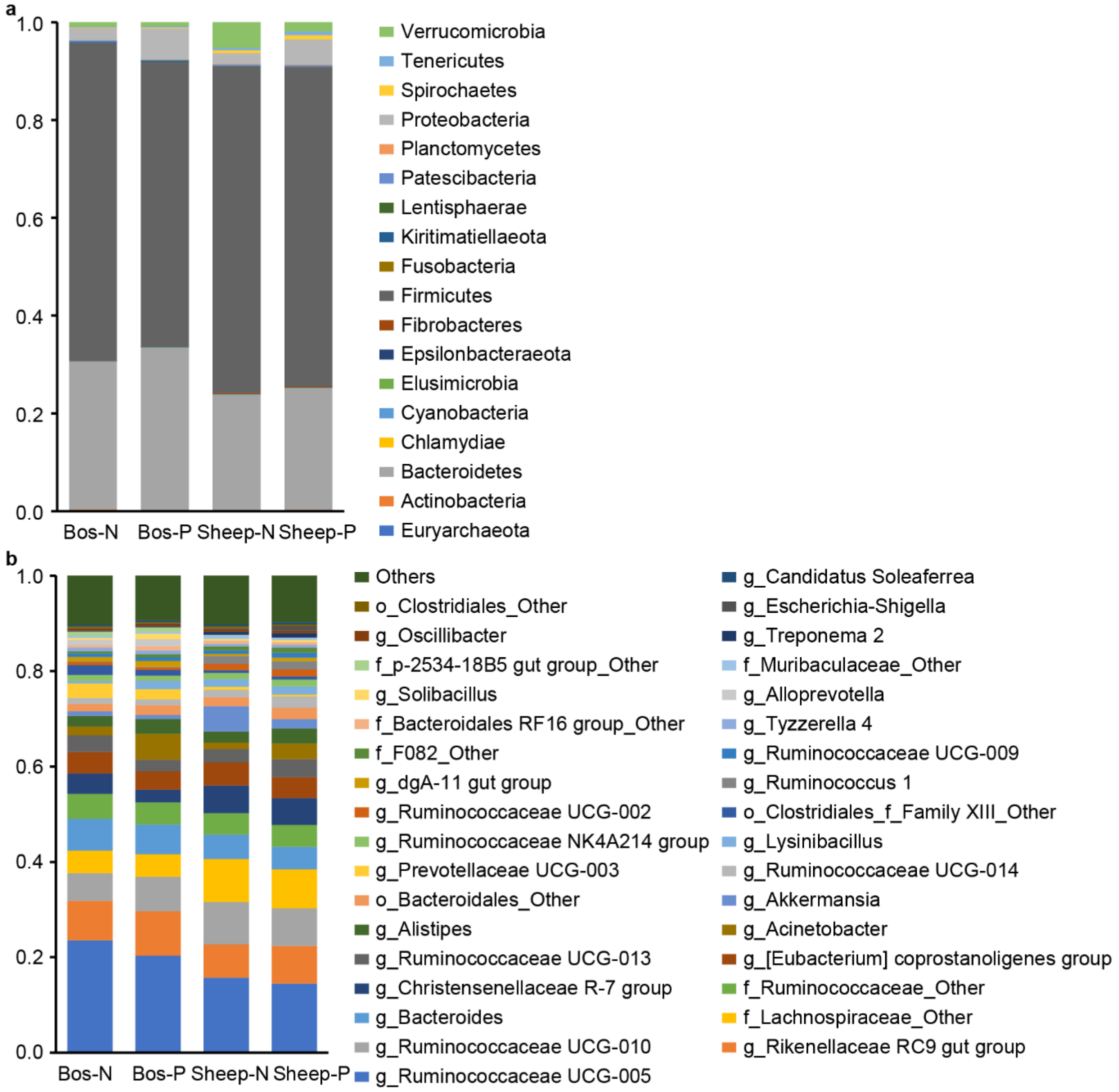

FIG S6 Microbial biomarkers of GIN positive and negative groups screened by random forest (RF) at genus level. The bacterial genera were identified by applying RF classification of the relative abundance of the fecal microbiota between GIN positive and negative groups. The curves represented the 10-fold cross-validation to determine the credible number of biomarkers. Biomarker taxa are ranked in descending order of importance to the accuracy.

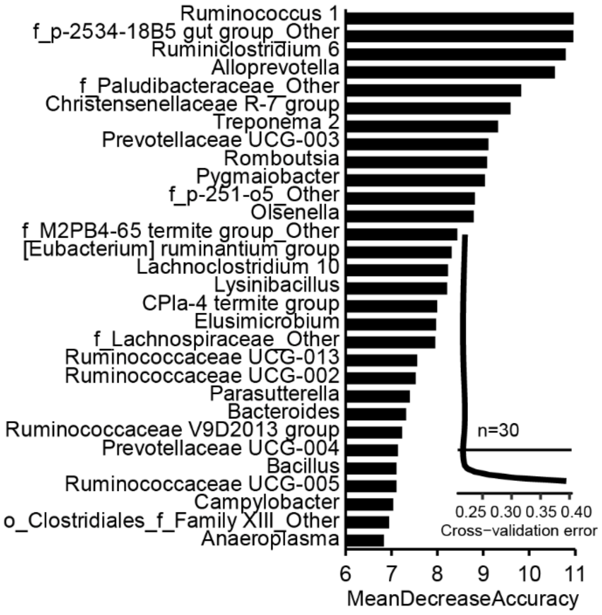

FIG S7 The assessment of optimal cluster number. The optimal cluster number calculated with Jensen–Shannon distance (JSD) using the Calinski–Harabasz (CH) index.

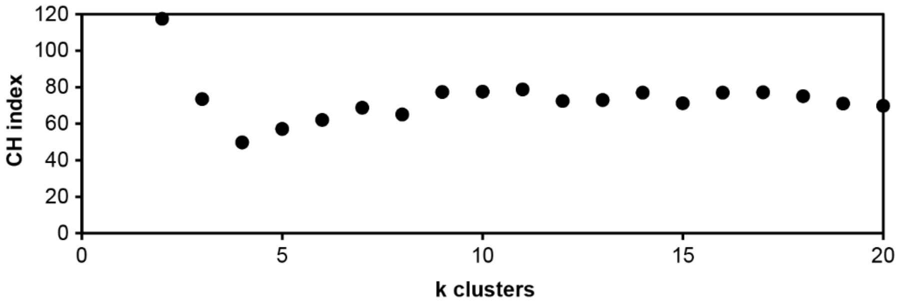

FIG S8 Distributions of the relative abundance of the driving genera of enterotypes. The plots showed the observed distributions of (a) *Ruminococcaceae UCG-005* and (b) *Acinetobacter* using a frequency distribution histogram with a density curve.

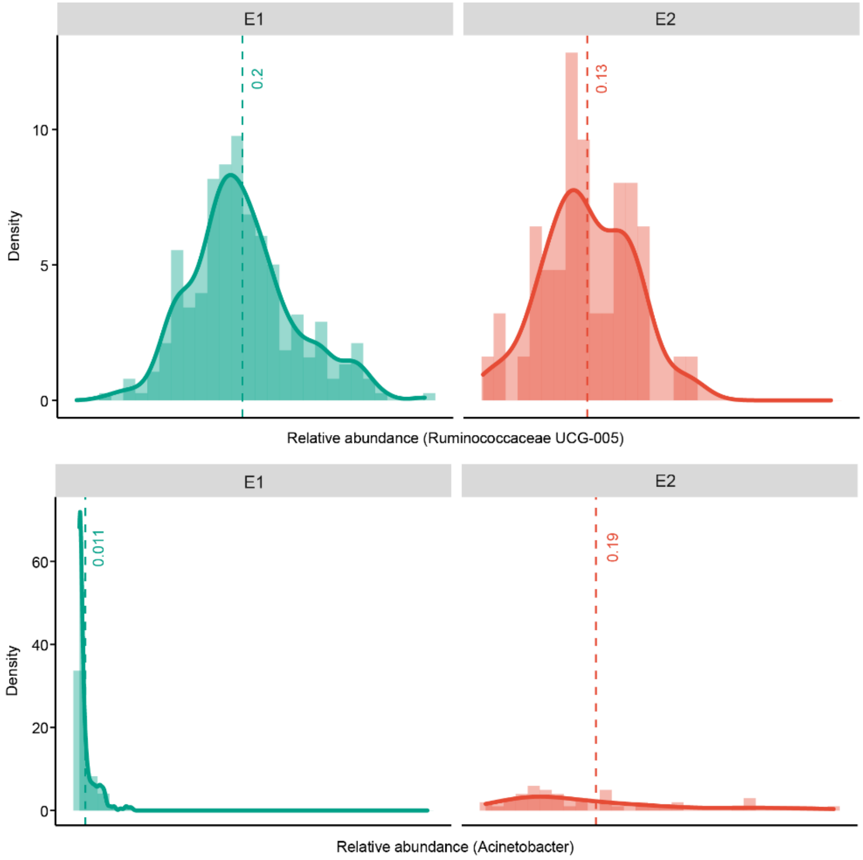

Supplement: Supplemental file 1 — Supplemental material. Download spectrum.00021-22-s0001.pdf, PDF file, 0.9 MB [file spectrum.00021-22-s0001.pdf]
